# Gigavalent Display of Proteins on Monodisperse Polyacrylamide Hydrogels as a Versatile Modular Platform for Functional Assays and Protein Engineering

**DOI:** 10.1021/acscentsci.2c00576

**Published:** 2022-08-01

**Authors:** Thomas Fryer, Joel David Rogers, Christopher Mellor, Timo N. Kohler, Ralph Minter, Florian Hollfelder

**Affiliations:** † Department of Biochemistry, University of Cambridge, 80 Tennis Court Road, Cambridge CB2 1GA, United Kingdom; ‡ Antibody Discovery and Protein Engineering, R&D, AstraZeneca, Milstein Building, Granta Park, Cambridge CB21 6GH, United Kingdom

## Abstract

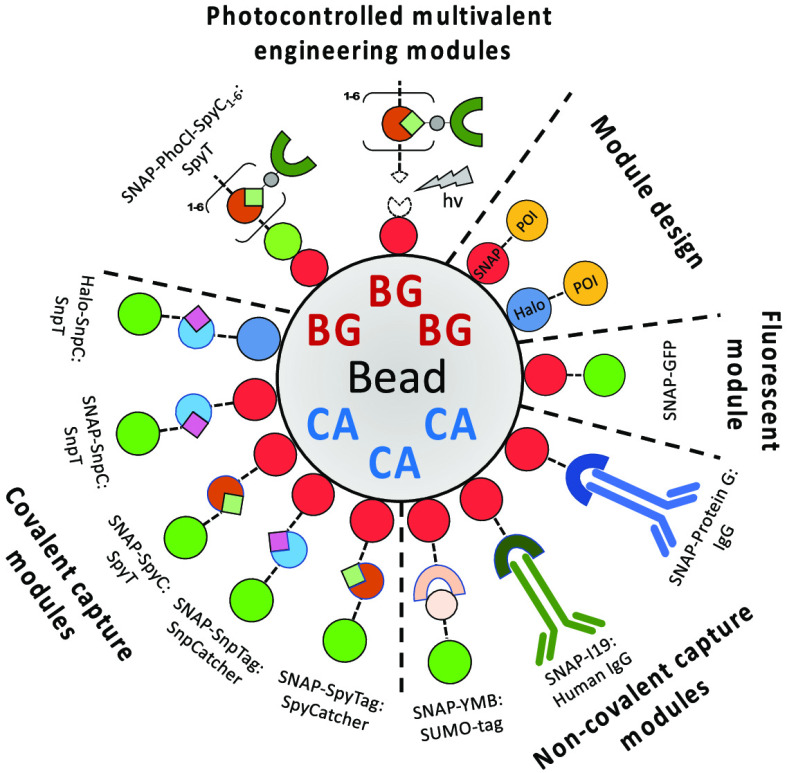

The assembly of robust, modular biological components into complex functional systems is central to synthetic biology. Here, we apply modular “plug and play” design principles to a solid-phase protein display system that facilitates protein purification and functional assays. Specifically, we capture proteins on polyacrylamide hydrogel display beads (PHD beads) made in microfluidic droplet generators. These monodisperse PHD beads are decorated with predefined amounts of anchors, methacrylate-PEG-benzylguanine (BG) and methacrylate-PEG-chloroalkane (CA), that react covalently with SNAP-/Halo-tag fusion proteins, respectively, in a specific, orthogonal, and stable fashion. Anchors, and thus proteins, are distributed throughout the entire bead volume, allowing attachment of ∼10^9^ protein molecules per bead (⌀ 20 μm) —a higher density than achievable with commercial surface-modified beads. We showcase a diverse array of protein modules that enable the secondary capture of proteins, either noncovalently (IgG and SUMO-tag) or covalently (SpyCatcher, SpyTag, SnpCatcher, and SnpTag), in mono- and multivalent display formats. Solid-phase protein binding and enzymatic assays are carried out, and incorporating the photocleavable protein PhoCl enables the controlled release of modules via visible-light irradiation for functional assays in solution. We utilize photocleavage for valency engineering of an anti-TRAIL-R1 scFv, enhancing its apoptosis-inducing potency ∼50-fold through pentamerization.

## Abstract 2

“Plug and play” display of up to ∼10^9^ protein molecules attached to monodisperse beads affords control over valency and release, creating a platform for analysis and use of proteins.

## Introduction

The analysis of proteins and their use as therapeutics,^1^ enzymes in biocatalysis^2^ and bioremediation,^3^ growth factors for tissue culture,^4^ or targets for binder discovery campaigns^5^ is often facilitated by the ability to capture, maintain, and manipulate proteins on biocompatible surfaces. Protein solid-phase immobilization is critical to many bioassays (e.g., ELISA^6^ and SPR^7^ for investigating protein:protein interactions) as it enables washing, modification, or rebuffering steps and interfaces with robotic workflows, using the protein attachment to handle the protein for testing in assays or for direct analyses. Industrial-scale biocatalysis can be enhanced by the sequestration/immobilization of valuable enzymes^2^ in continuous flow biocatalysis,^8,9^ while also offering potential synergistic effects through the colocalization of specific enzymes.^10^ Proteins immobilized on surfaces have also emerged as useful therapeutic agents, enhancing *in vivo* half-life and providing extra control over drug delivery (both temporally and spatially).^1,11^ Despite the demonstrated utility of immobilized proteins across multiple fields, the methods of immobilization are highly diverse and typically bespoke. Protein function and stability can be impacted by surface effects (observed, e.g., for immobilized targets in phage display^5,12^ and enzymes in biocatalysis^13−15^): spectroscopic interference (such as autofluorescence^16^) can negatively affect bioassay sensitivity; the stoichiometry and strength of attachment are variable on heterogeneous solid-phase supports; and there can be batch-to-batch variation that hampers the development of robust and reproducible protocols.

To simplify engineering of protein capture across a variety of fields, e.g., protein engineering, biocatalysis, and therapeutic protein delivery, it is desirable to develop new technologies that address many of the aforementioned issues. Ideally, the technology would be versatile, controllable, and robust (minimizing the customization and optimization required for each new application); the user would have precise control over protein capture density as well as the size of the immobilization matrix; and the capture mechanism and matrix would not themselves affect protein functionality or interfere with the envisaged application. To enhance the accessibility of such a technology, a core design principle should be that of “plug and play” modular components that are easy to produce and engineer. In the context of protein capture and manipulation, this should empower a researcher using this system to focus their efforts on engineering complex protein-based systems rather than having to extensively validate or troubleshoot the individual base components. Robust (i.e., stable, both over time and under diverse conditions) protein capture through the trusted modular assembly of “plug and play” components thus provides molecular Lego that simplifies the design of, e.g., synthetic biology^17^ experiments, just as click chemistry^18,19^ has made aspects of synthetic chemistry generalizable, versatile, and easy to use. Such robust molecular biology tools have arisen at the interface of protein engineering and synthetic biology in recent years, notably SpyCatcher, among others,^20,21^ as a plug and play tool for post-translational valency engineering and protein purification;^22^ photocontrollable proteins such as PhoCl^23^ for the spatiotemporal control of protein release via light-induced protein backbone cleavage;^24^ or new highly stable and versatile protein recognition elements such as the ALFA-tag system.^25^ While extant protein immobilization methods (e.g., Ni-NTA, streptavidin, protein A/G, and chemical cross-linking) have been used successfully with such technologies (e.g., for purification of recombinantly expressed versions), no single system incorporates all of the desired traits that are required of a system suitable for applications across a wide range of fields: versatility, controllability, and robustness. We consider that the lack of such a technology curtails the engineerability, and thus possible applications, of protein-based systems.

Of the surfaces functionalized with proteins, hydrogels^26^ are an increasingly important matrix for biological applications due to their biocompatibility (permeability, adjustable stiffness, and low cytotoxicity). They have found use in single-cell transcriptomics,^27^ mammalian cell culture,^28^ or *in vivo* drug delivery devices^1^ or as artificial cells.^29^ In particular, surface effects can be minimized by the absence of a hydrophobic surface that can lead to protein denaturation. Hydrogels functionalized with protein have been demonstrated utilizing a diverse array of capture methods (e.g., anti-His-tag aptamers,^29^ molecular imprinting,^30^ click chemistry,^24^ and copolymerization with acrylamide^31^ or through disulfide bond formation^32^), yet no method has been demonstrated that fulfils the criteria of versatility, controllability, and robustness.

Here, we introduce a platform that incorporates robust, covalent, site-specific protein capture within a hydrogel matrix in a highly modular fashion that offers stability, versatility, and accessibility. Using precisely defined, highly specific, orthogonal, and covalent protein capture within a hydrogel matrix, a suite of “plug and play” secondary functionality modules were developed that achieve noncovalent or covalent capture of defined proteins, at precisely defined valencies, and with photocontrollable release of assembled proteins into solution. We seek to demonstrate the utility of this platform and associated tools through their application to protein binding studies, enzymatic assays, phenotypic cellular assays, and therapeutic protein engineering. These examples demonstrate the platform’s versatility, as only minimal engineering is required to repurpose the system for a new application.

## Results and Discussion

### Design of Polyacrylamide Hydrogels with Titratable Protein Capture

**Figure 1:**
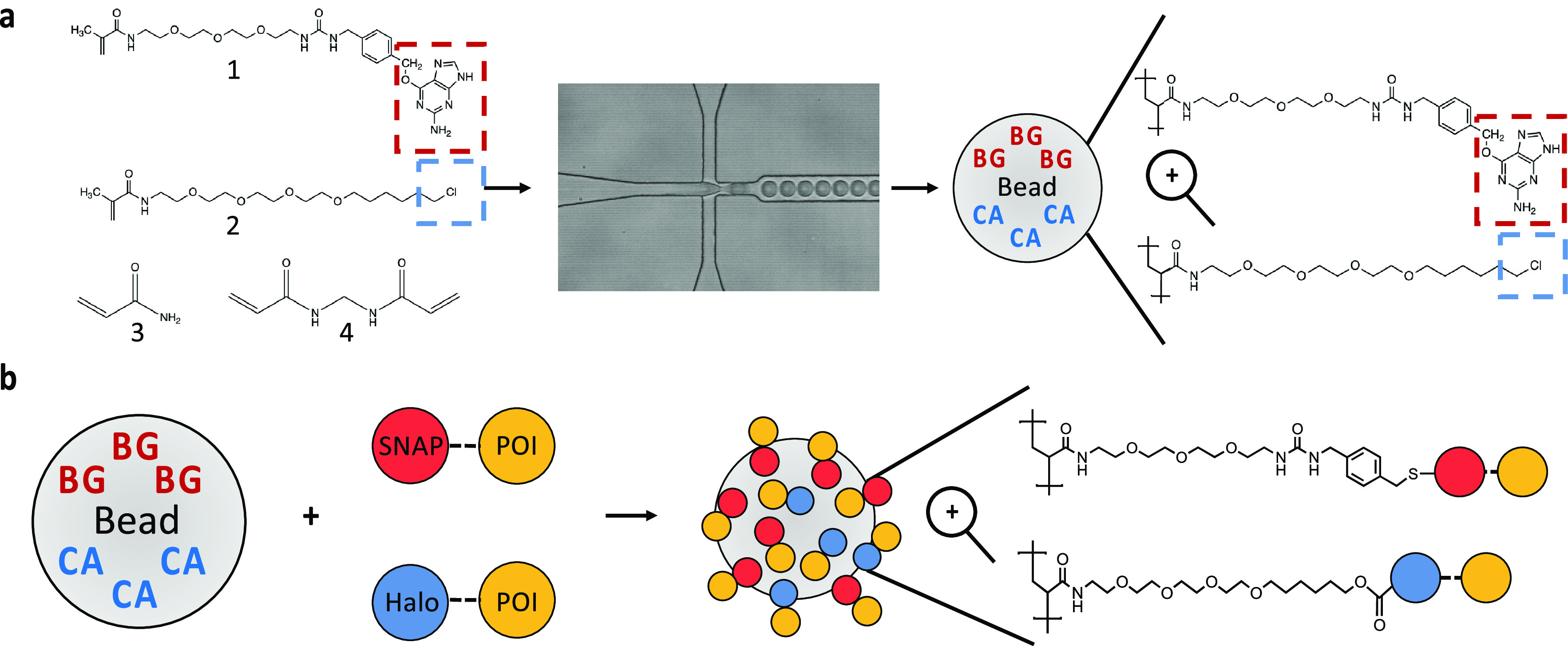
Modular polyacrylamide hydrogel display. (a) Monodisperse polyacrylamide hydrogel beads are made through the encapsulation of monomers [(1) methacrylate-PEG-benzylguanine (BG), (2) methacrylate-PEG-chloroalkane (CA), (3) acrylamide, (4) bis-acrylamide] with polymerization-inducing catalysts using droplet-based microfluidics. Upon de-emulsification, BG (red) and/or CA (blue) are retained within each bead due to copolymerization with the hydrogel backbone. (b) Hydrogel beads can then be orthogonally functionalized with SNAP- or Halo-tag fusion proteins (red and blue, respectively) through covalent reaction with their respective copolymerized small molecule ligands (BG/CA).

Synthesized from components found in most molecular biology laboratories (e.g., to make SDS-PAGE gels) and with a proven reliability of polymerization, polyacrylamide hydrogels are easy to use and have readily engineerable mechanical properties (e.g., stiffness and porosity^33^). Alongside their widely known applications as protein separation reagents, polyacrylamide hydrogels have already taken a role as biocompatible scaffolds for the delivery of reagents in microfluidic single-cell transcriptomic workflows,^27^ in cell culture support matrices (with a particular focus on investigating mechanoelastic effects^34^), and as *in vivo* drug delivery devices.^35,36^ Despite the proven interest in polyacrylamide hydrogels, no simple, stable, and modular technology exists for their functionalization with proteins. Polyacrylamide hydrogels consist of chains of monomers of acrylamide that are cross-linked by bis-acrylamide in stable polymers. Through the variation of acrylamide/bis-acrylamide ratios, hydrogels of different pore sizes and mechanical properties can be brought about as desired for an intended application. Secondary properties can also be engineered in, such as dissolution in response to redox, protease, or pH cues. To enable the capture of proteins, we copolymerized acrylamide and bis-acrylamide monomers with methacrylate-modified small molecule ligands [methacrylate-PEG-benzylguanine (BG) and methacrylate-PEG-chloroalkane (CA); Figure 1a]. These ligands act as suicide substrates for SNAP-tag^37^ and Halo-tag,^38^ respectively, and their copolymerization throughout the hydrogel enables completely covalent capture of an array of modular building blocks expressed as fusion proteins to these tags (Figure 1b). SNAP-tag and Halo-tag are both well-established protein tags used across biological fields and can be expressed in bacterial, yeast, and mammalian cell lines.^37^ Notably, SNAP-tag and Halo-tag react orthogonally with their respective ligands (BG and CA) and have already been used to capture proteins on surfaces,^39,40^ yet this orthogonality has not been fully exploited for protein capture on bifunctional surfaces and “plug and play” modules, for protein engineering and assay design have not been developed.

**Figure 2:**
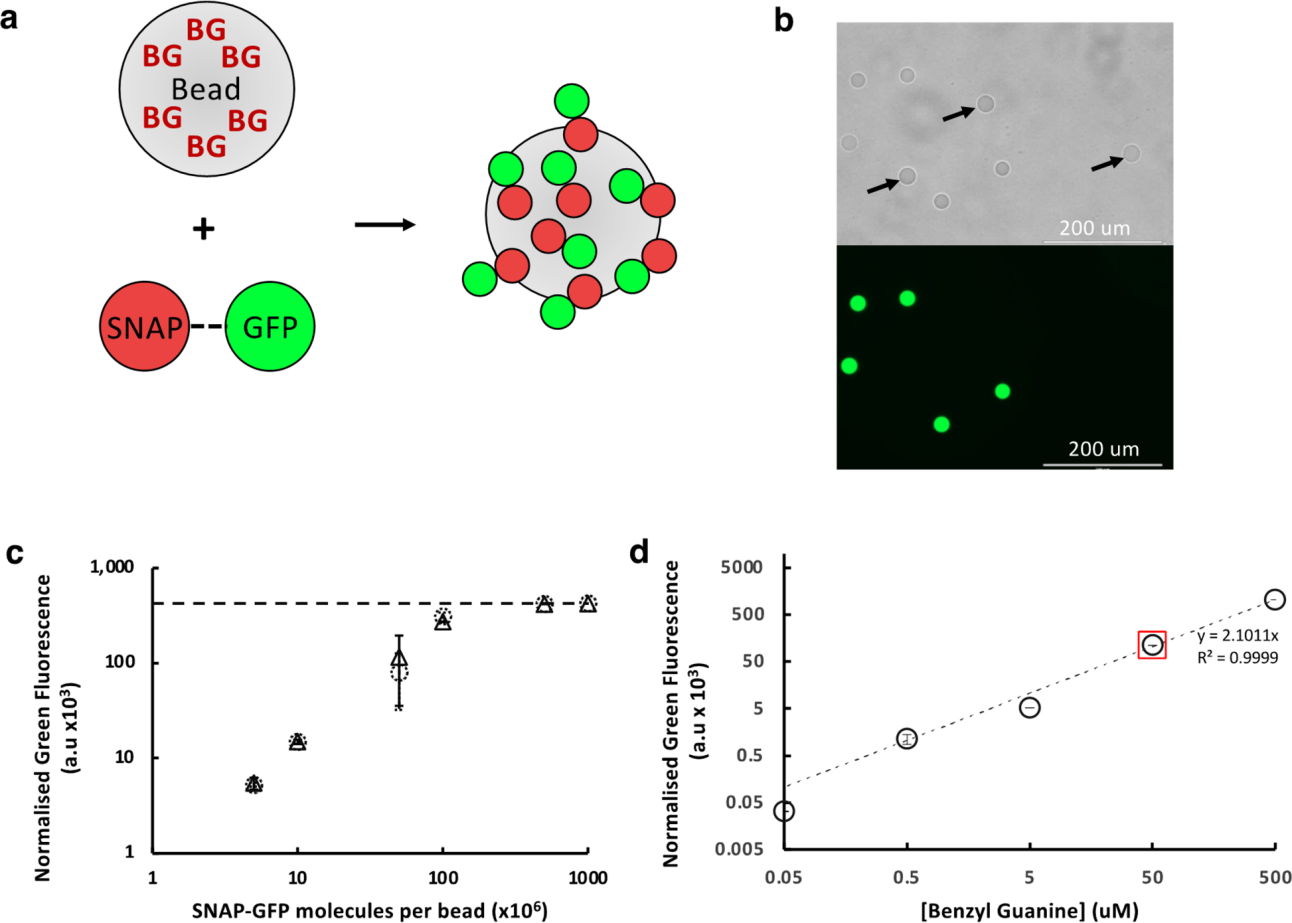
Specific, stable, and titratable protein capture on polyacrylamide hydrogel beads. (a) BG-functionalized hydrogel beads are incubated with SNAP-GFP, leading to the covalent capture of SNAP-GFP on-bead. (b) 20 μm PHD beads ±50 μM BG were mixed 50:50 and incubated with SNAP-GFP followed by washing and imaging (top panel bright-field, bottom panel GFP channel) to detect specific GFP attachment. Scale bar: 200 μm. Arrows in the bright-field image indicate nonfunctionalized beads, demonstrating very low nonspecific protein binding. (c) 100 000 20 μm, 50 μM BG PHD beads were incubated with defined numbers of SNAP-GFP molecules per bead overnight, washed, and analyzed by flow cytometry. The saturation point, i.e., where the addition of extra SNAP-GFP does not lead to an increase in on-bead fluorescent signal (dashed line), corresponds to a density of ∼150 million attached proteins per bead. Black triangles indicate boiled beads; open dashed circles indicate beads handled according to our standard procedure (see the Experimental Section). (d) Five sets of 20 μm PHD beads were prepared with the indicated BG loading. All were incubated with an excess of SNAP-GFP, washed, and analyzed by flow cytometry. The red square highlights the 50 μM BG beads used in panel c that captured 1.5 × 10^8^ SNAP-GFP molecules per bead; the near-perfect correlation (fitted to a linear model, with an intercept at 0 due to background signal subtraction; displayed as a double logarithmic plot to capture the wide range of concentrations) between [BG] and green fluorescence shows that a valency range of 10^5^–10^9^ per bead was achieved (for 0.05, 0.5, 5, 50, and 500 μM BG beads, respectively). Data are the mean of triplicates, normalized to the background signal of PHD beads lacking BG. The error displayed is the standard deviation.

We prepared methacrylate-PEG-benzylguanine/methacrylate-PEG-chloroalkane by reacting methacrylate-NHS ester with amine-PEG-benzylguanine/amine-PEG-chloroalkane overnight in a simple click reaction and achieved a near-quantitative yield (>90%, as measured by HPLC, Figure S1.1 and Table S1.1). The products of these reactions can then be directly used for copolymerization into polyacrylamide hydrogels, so we subsequently generated BG-functionalized monodisperse beads of 20 μm diameter (Ø) using droplet-based microfluidics at ∼8 kHz (enabling production of 29 million beads per hour). 20 μm beads are readily compatible with downstream analysis technologies such as flow cytometry and represent a readily visualized size for microscopy. However, a variety of sizes can be made through the use of different chip geometries and flow rates as the particle size is controlled by the size of the microdroplet it is polymerized within, e.g., in the InDrop technology (63 μm),^27^ or in a study by Abate et al. (30 μm),^41^ and alternative technologies can even enable nanometer-scale polyacrylamide particles to be made.^35,36^ Upon de-emulsification, BG-functionalized hydrogel beads can be incubated with SNAP-tag fusion proteins (such as SNAP-GFP) for covalent capture (Figure 2a). A key feature of polyacrylamide hydrogels is their low levels of nonspecific interactions with proteins, thus enabling the highly specific capture of defined proteins. This specific protein capture is exemplified in Figure 2b: only beads functionalized with BG are able to capture SNAP-GFP, and there is little-to-no nonspecific binding to nonfunctionalized polyacrylamide beads. Polyacrylamide hydrogel display (PHD) beads can also be made entirely *without* the use of microfluidics, by vortexing the aqueous monomer solution with surfactant-containing oil (the same compositions as for microfluidics) to create polydisperse emulsions. These polydisperse hydrogel beads vary somewhat in size but still function as programmed for capture of, e.g., SNAP-GFP (Figure S1.2), thus enabling their use as protein-capture matrices by researchers without a microfluidic setup in many of the same applications as demonstrated for monodisperse beads within this Article.

Next, we sought to quantify the capacity of on-bead coupling. When incubating beads (Ø 20 μm, 50 μM BG) with increasing amounts of SNAP-GFP, we observed asymptotic saturation of the fluorescence signal (after washing of beads) at ∼5 × 10^8^ SNAP-GFP molecules per bead, and we found this binding behavior to be highly conserved even when beads are boiled (100 °C for 10 min) before protein capture, demonstrating the high stability of this system (Figure 2c). In order to estimate the number of molecules required to saturate a bead more accurately, we extrapolated the linear part of our saturation curve up to the asymptote. This calculation suggests that ∼1.5 × 10^8^ SNAP-GFP molecules per bead are bound at saturation [equal, within experimental error, to the calculated 1.3 × 10^8^ BG molecules per bead (Ø 20 μm, 50 μM BG bead; Figure S1.3a)]. Such high occupancy levels of immobilized proteins exceed those achieved with magnetic beads that bind proteins on their surface by 3 orders of magnitude^42^ (M-280 streptavidin Dynabeads, ∼6.6 × 10^5^ IgG molecules per bead, Figure S1.3b). The difference can be ascribed to the voluminal nature of protein capture, wherein not only is the bead’s surface functionalized, but also its interior, as demonstrated by the uniform distribution of fluorescence in confocal images of the beads (Figure S1.4a). Our confocal data also confirmed the long-term stability of the BG-functionalized PHD beads, with an estimated 1/3 of binding capacity being retained even after 3 years of storage at 4 °C (Figure S1.4b). In addition to the high levels of protein capture, it is also possible to precisely control the amount of captured protein by changing the concentration of BG monomers included in the hydrogel polymerization mix. When the concentration of BG in the initial one-pot prepolymerization acrylamide mix is varied, the amount of SNAP-GFP captured varies correspondingly; display densities spanning at least 5 orders of magnitude can be brought about at will, and an estimated 1.5 × 10^9^ molecules are bound when using 500 μM BG (Figure 2d), demonstrating gigavalent capture. The demonstrated stability of the matrix and the ability to precisely control protein capture density across multiple orders of magnitude, alongside the previously documented ability to modulate the size and mechanical properties of the polyacrylamide hydrogel, highlight the versatility and precise tunability of the system.

### Specific Protein Capture via Noncovalent Secondary Capture Modules

**Figure 3:**
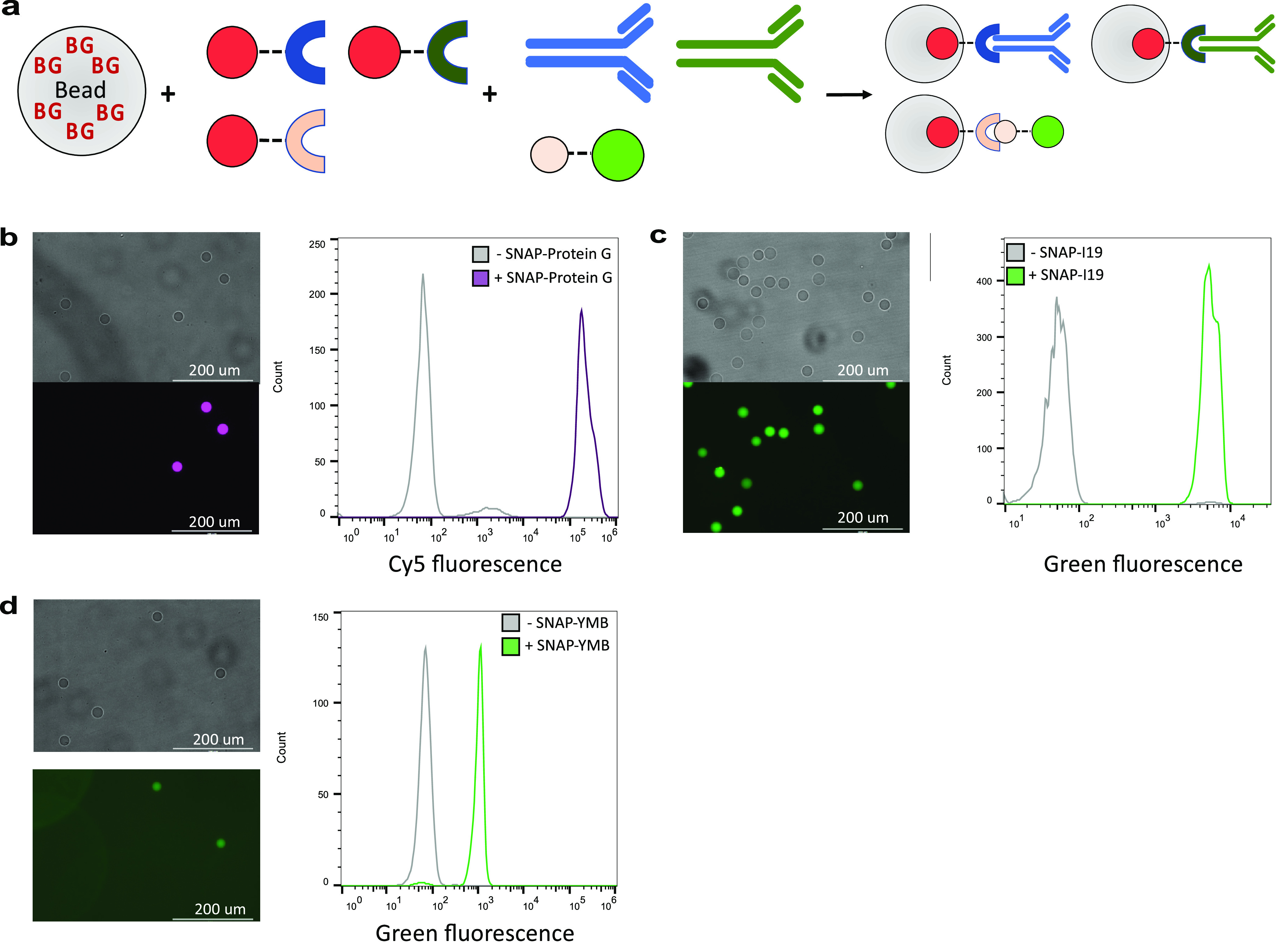
Versatile capture of modular building blocks for specific protein capture. (a) BG-functionalized hydrogel beads can be used to covalently immobilize secondary capture modules (as SNAP-tag fusions; SNAP shown in red) that are specific for a desired target protein. PHD beads (Ø 20 μm; 50 μM BG) ± their respective modules: (b) SNAP-Protein G, (c) SNAP-I19, or (d) SNAP-YMB were incubated with their target proteins (Mouse IgG-iFluor 647/blue, human IgG1-AlexaFluor 488/green, SUMO tag-GFP/bright green, respectively) for 1 h, washed, and analyzed by both fluorescent microscopy (left-hand panels, beads ± SNAP-tag capture module were mixed 50:50 and imaged together) and flow cytometry (right-hand panels, beads ± SNAP-tag capture module analyzed separately and superimposed). Scale bars represent 200 μm.

Having established the ability to functionalize polyacrylamide hydrogels for highly stable and controllable protein capture, we next sought to establish “plug and play” functional modules for protein capture that would enable researchers to use this system in a simple manner, while taking advantage of the key features of the system (stability, controllability, and specificity). While we have already demonstrated the direct capture of a protein of interest as a SNAP-tag fusion (Figure 2), it is also possible—and greatly enhances the utility of the PHD technology as an engineering tool—to use specific secondary capture modules (e.g., affinity reagents fused to SNAP-tag) to assemble proteins of interest in bead (Figure 3a). Importantly the use of such “plug and play” secondary capture modules enables a researcher to capture specific proteins that are not themselves SNAP-tagged, enabling the capture of either nontagged native proteins (e.g., IgG) or recombinantly expressed proteins fused to smaller, less intrusive tags (e.g., SpyTag and SnpTag). As the base bead remains the same (20 μm, 50 μM BG), its desired functionality can be altered simply by choosing which secondary capture module to initially capture on-bead. To demonstrate this principle, we fused several secondary capture modules to SNAP-tag for immobilization: SNAP-Protein G for mouse IgG capture (Figure 3b); SNAP-I19,^43^ an antihuman IgG DARPin, for human IgG capture (Figure 3c); and SNAP-YMB,^44^ an anti-SUMO monobody, for capture of SUMO-GFP (Figure 3d). In all figures, the specificity of capture is demonstrated in control experiments by the lack of fluorescence on beads not functionalized with the respective secondary capture module. These secondary capture modules are all readily expressed in bacteria, obviating the need to buy expensive affinity reagents for desired applications. Further secondary capture modules can be designed based on published sequences of affinity reagents, or freshly developed through *de novo* discovery techniques such as phage display, and assembled in a modular fashion using, e.g., Gibson assembly (details in Figure S1.5, Experimental Section S2.3). We demonstrate these protein capture examples not as tasks that can uniquely be completed with this system but as routine applications that could subsequently benefit from the associated unique attributes of stability, controllability, and specificity.

### Modular and Orthogonal Programming of Bead Functionality via Covalent Secondary Capture Modules

**Figure 4:**
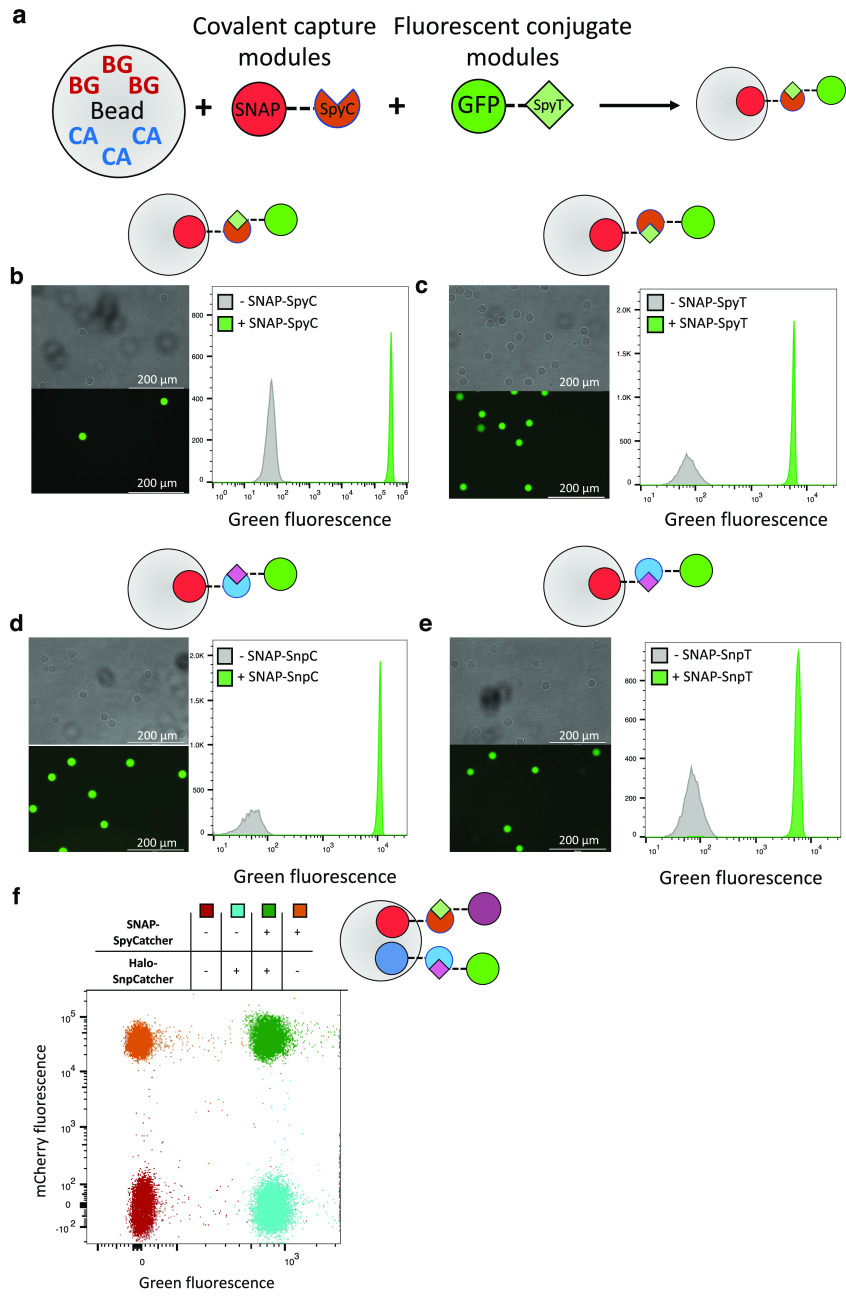
Versatile, orthogonal, and covalent capture of target proteins. (a) BG/CA-functionalized hydrogel beads can be used to covalently immobilize secondary covalent capture modules (as SNAP-tag or Halo-tag fusions) that specifically react with their partner tag. Throughout the figure, relevant protein domains are indicated as SNAP-tag (red circle), Halo-tag (blue circle), SpyCatcher (orange circle with 1/4 cut-out), SnpCatcher (blue circle with 1/4 removed), SpyTag (green diamond), SnpTag (pink diamond), GFP (green circle), and mCherry (purple circle). Monofunctionalized PHD beads (50 μM BG, Ø 20 μm) were incubated ± SNAP fusion proteins: (b) SNAP-SpyCatcher; (c) SNAP-SpyTag; (d) SNAP-SnpCatcher; or (e) SNAP-SnpTag. These beads were then mixed and incubated with their respective target proteins [(b) GFP-SpyTag; (c) GFP-SpyCatcher; (d) GFP-SnpTag; and (e) GFP-SnpCatcher] for 1 h, washed, and analyzed by both fluorescent microscopy (left-hand panels) and flow cytometry (right-hand panels). Scale bars represent 200 μm. (f) Bifunctionalized PHD beads (50 μM BG, 50 μM CA, Ø 20 μm) were incubated ± SNAP-SpyCatcher and/or Halo-SnpCatcher; these beads were subsequently incubated with both GFP-SnpTag and mCherry-SpyTag for 1 h, washed, and analyzed by flow cytometry.

Next, to enable *covalent* immobilization of proteins of interest, we designed additional secondary capture modules as both SNAP- and Halo-tag fusions to the suite of SpyCatcher/SpyTag and SnpCatcher/SnpTag technologies^45^ (Figure 4a). These protein pairs form an isopeptide bond under standard biological reaction conditions and have already been applied widely to the modular engineering of proteins (e.g., vaccine design,^46^ protein cyclization for enzyme engineering,^47^ and multivalent and multifunctional protein assembly^48^). In this work we use SpyCatcher ΔNC^49^ (a deimmunized SpyCatcher truncation) and SpyTag002^50^ (an evolved SpyTag with enhanced reaction kinetics). Importantly, the two pairs react orthogonally (as do SNAP-tag and Halo-tag), enabling the specific modular construction of multifunctional beads with relative ease. While SpyTag and SnpTag have both been incorporated into hydrogel frameworks previously (PEG-functionalized^31^ or all-protein hydrogels^51^), the versatility of these systems is limited compared to that displayed here in which any and all arrangements of protein pairs can be assembled on-bead (Figure 4b–e) simply by exchanging the covalent secondary capture module first captured on-bead. Due to the orthogonality of the four protein capture technologies employed (SNAP-tag, Halo-tag, SpyCatcher, and SnpCatcher), specific capture of target proteins can be programmed by simply functionalizing beads ± any desired component. In Figure 4f, we demonstrate the highly controlled capture of GFP-SnpT/mCherry-SpyT based solely upon the previous functionalization of beads with/without SNAP-SpyCatcher/Halo-SnpCatcher. These beads now exhibit programmed bifunctionality (both GFP and mCherry fluorescence) and serve to demonstrate the versatility, modularity, and orthogonality of the PHD technology. As before, researchers can design and express further capture modules and functionalities with relative ease through the use of modular Gibson assembly.

### Application of PHD Beads to Bioassays: Protein–protein Interactions, Enzymatic Catalysis and Bacteriolysis

**Figure 5:**
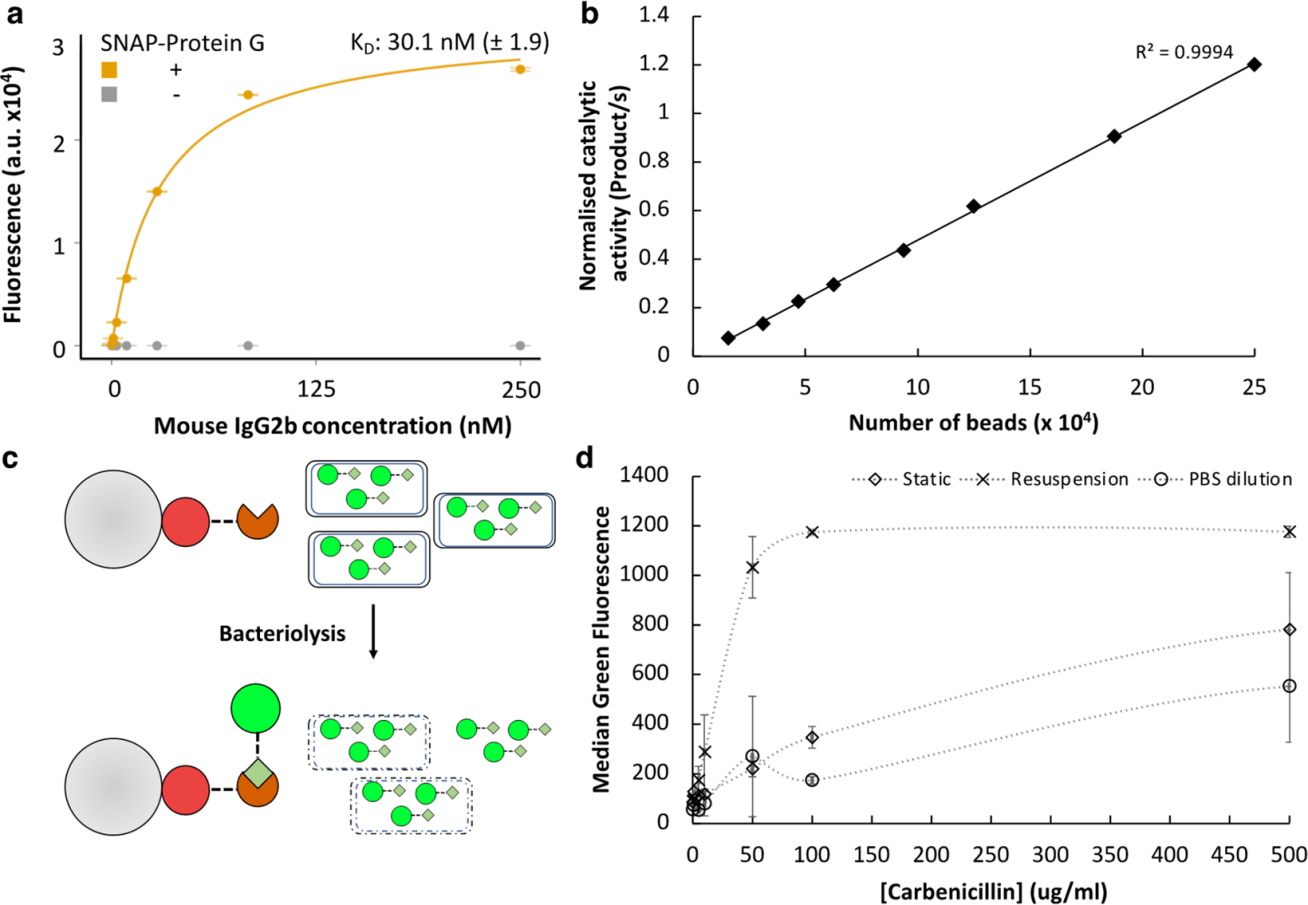
PHD beads in designed functional bioassays. (a) PHD beads (50 μM BG, Ø 20 μm) functionalized with/without SNAP-Protein G were incubated with a titration series of fluorescently labeled mouse IgG2b at room temperature with rolling for the indicated times. Beads were recovered, washed, and analyzed by flow cytometry. Data are the mean of triplicates, and the curve was fitted to the following equation: fluorescence = (fluorescence_max_ × [IgG])/(*K*_D_ + [IgG]). (b) *In vitro*-expressed P91-SpyTag was captured on-bead, washed, and incubated with 50 μM substrate [fluoresceine-di(diethylphosphate)] in a 100 μL volume. Bead number per well was varied as indicated. The initial 90 min of reaction was used to calculate the catalytic activity. Data are presented normalized to nonfunctionalized beads to control for background hydrolysis of the substrate. (c) Overview of bacteriolysis sensor design. PHD beads functionalized with the SNAP-SpyC covalent capture module are incubated with bacterial cells expressing GFP-SpyT. Only upon lysis will the GFP-SpyT be released into solution and be able to be captured on the sensor beads. (d) *E. coli* cells expressing GFP-SpyTag were grown overnight with induction of protein expression. Static cultures (blue), cultures resuspended in fresh culture media (orange), and static cultures diluted 1:1 with PBS (gray) were incubated with a range of carbenicillin concentrations for 90 min at 37 °C in triplicate. Cultures were pelleted, and the supernatant was transferred to incubate with SpyCatcher-functionalized PHD beads for 60 min. Beads were washed twice and then analyzed by flow cytometry.

Due to the modularity and robustness of the PHD technology, it is facile to design and implement bioassays. We demonstrate this for assaying protein–protein interactions—an extremely common bioassay which is key to understanding basic molecular interactions (e.g., in the development of protein-based therapeutics)—by carrying out an investigation into the binding affinity of SNAP-Protein G for a mouse IgG subtype (Figure 5a). We incubated SNAP-Protein G-functionalized beads with a titration series of fluorescently labeled mouse IgG2b, before washing away unbound IgG and measuring the amount of binding by flow cytometry. This experiment bears close resemblance to those designed for yeast surface display-mediated measurements of binding affinity^52^ that have proven to compare favorably to the “gold-standard” method of surface plasmon resonance. An affinity of 30.1 nM was calculated, in close accordance with published data^53^ on Protein G binding to mouse IgG (41.5 nM, we note that data is binding to total mouse IgG rather than an individual subclass).

Having demonstrated the utility of PHD beads for assaying protein–protein interactions, we wished to also highlight their suitability for simplifying and improving the quality of high-throughput assays. SNAP-SnpTag was captured directly from bacterial cell lysate and probed by subsequent incubation with GFP-SnpCatcher. A minimal volume of 2× concentrated cell lysate (1 μL) corresponding to ∼2 μL of culture volume (1/500 of the largest volume tested) was found to already saturate 50 000 20 μm, 50 μM BG beads (Figure S1.6). Direct capture of a protein of interest from cell lysate obviates the need for a separate purification step, while the precise control over protein capture through user-controlled BG concentration and bead number effectively achieves expression level normalization for a subsequent assay. Protein expression, lysis, on-bead capture, and the subsequent assay (flow cytometry) were all carried out in a 96-deep-well plate format; combining the PHD beads with high-throughput, sensitive techniques such as flow cytometry creates a powerful platform with which multiple parameters (e.g., affinity and specificity) can be examined simultaneously, and assays can be multiplexed for even greater throughput.^54^

In addition to protein–protein interactions, another common form of bioassay is enzymatic catalysis, in which the accumulation of product or loss of substrate is followed over time. The immobilization of enzymes is of great interest for industrial biocatalysis^2^ and can also serve to provide a simple method of delivering a defined concentration of protein to a given assay—an important feature when comparing the activity of enzyme variants in a directed evolution experiment for instance. To demonstrate the precise control of enzyme concentration for use in a subsequent bioassay, we captured P91^55^-SpyTag, a phosphotriesterase, on SNAP-SpyCatcher-functionalized beads. The number of beads per reaction was varied and the accumulation of product followed by an increase in fluorescence signal (Figure 5b). A near-perfect linear relationship is seen between bead number per reaction and catalytic activity, highlighting two key points: first, that the captured enzyme remains functional on-bead and, second, that the quantity of enzyme delivered to an assay can be precisely controlled by the number of enzyme-functionalized beads delivered to that assay. In addition, as a proof-of-principle, this experiment also highlights the compatibility of PHD beads with the cell-free expression of proteins, as P91-SpyTag was expressed using PURExpress and directly captured on-bead from the *in vitro* expression reaction. Cell-free expression of proteins is now a well-established field^56^ with commercial products available and can enable the rapid, ultrahigh-throughput expression of even cytotoxic proteins.^57^

Next, to demonstrate that our platform’s applications are not limited to cell-free bioassays, we designed a microtiter plate- and flow cytometry-compatible sensor for bacteriolysis to facilitate the discovery of antibacterials (Figure 5c). PHD beads were first functionalized with the SNAP-SpyCatcher covalent capture module before being incubated with *Escherichia coli* which expressed GFP-SpyTag intracellularly and had been exposed to carbenicillin at a range of different concentrations (0–500 μg/mL) and under three different conditions: static culture; culture diluted 1:1 in PBS; and culture resuspended in fresh media (Figure 5d). Bacteriolysis is sensed by the release of GFP-SpyTag from lysed bacteria and its subsequent capture on SNAP-SpyC-functionalized PHD beads. These sensor beads can then be recovered and quantitatively analyzed by flow cytometry. We observed that resuspension of cells in fresh media was necessary for the maximal induction of bacteriolysis, and we further note that these results implicate carbenicillin (and/or related molecules) as an effective protein extraction reagent. The observation that resuspension is required for cell lysis is supported by the literature^58^ and a mechanistic understanding of how carbenicillin acts^59^ (as an inhibitor of transpeptidases required for cell wall biosynthesis).

### Valency Engineering and Photocontrolled Release of Antibody Drugs for Phenotypic Assays

**Figure 6:**
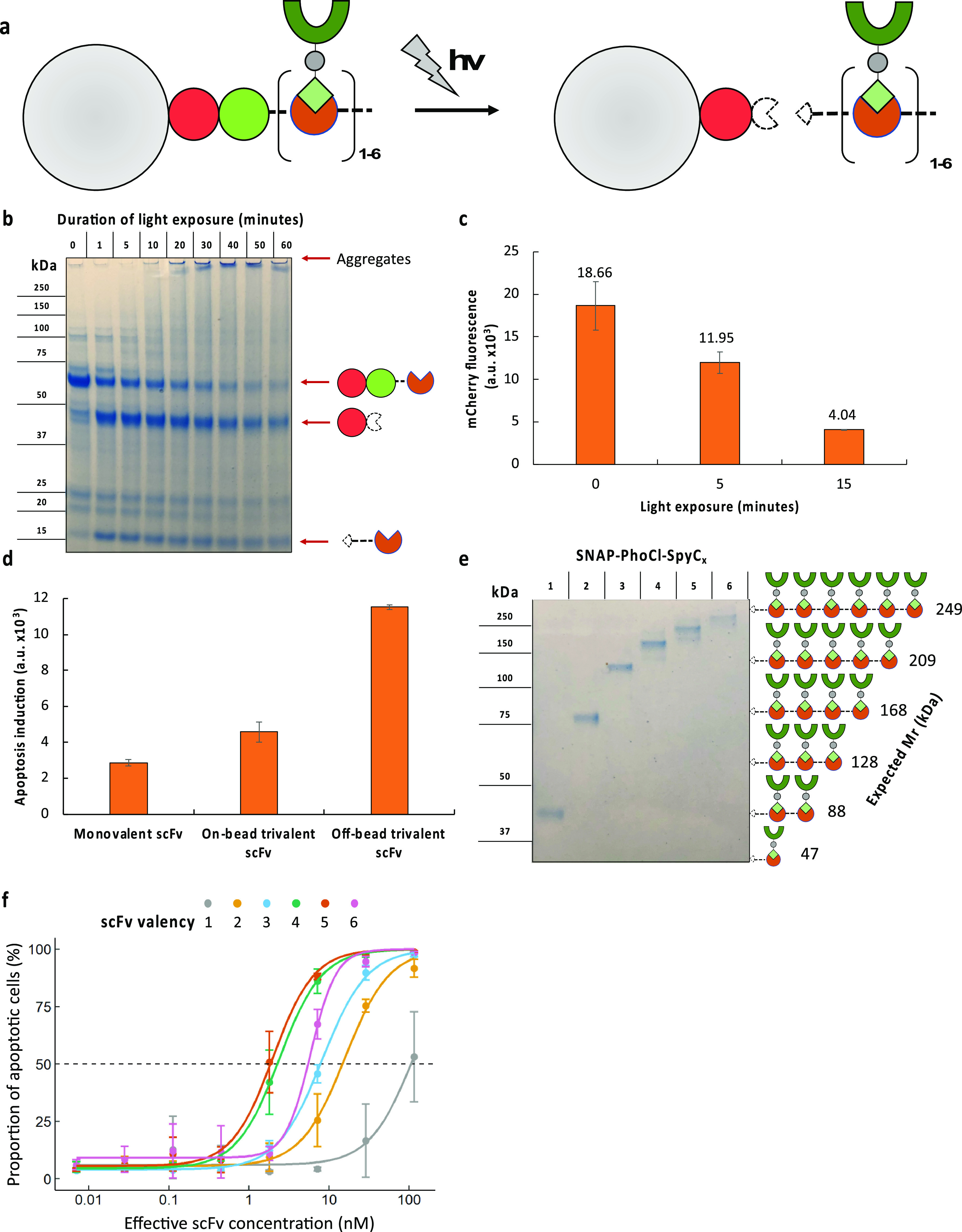
Photocontrolled valency engineering for antibody drug phenotypic assays. (a) Beads can be functionalized with the valency engineering covalent capture modules (SNAP-PhoCl-SpyC_1–6_) and subsequently used to capture SpyT-POI (here, scFv-SpyT). Upon exposure to 405 nm light, the PhoCl protein self-cleaves and releases the valency-modified assembly into solution. (b) SNAP-PhoCl-SpyC_1_ was exposed to 405 nm light for the indicated durations and the samples loaded on a denaturing SDS-PAGE gel for analysis of cleavage. (c) PHD beads functionalized with SNAP-PhoCl-SpyC_1_ were exposed to 405 nm light for the indicated durations. Beads were then washed and incubated with mCherry-SpyTag followed by flow cytometry. Data represent the mean of triplicates, and the fluorescence values are displayed above each bar. (d) 100 000 20 μm 50 μM BG beads for each sample were incubated with SNAP-PhoCl-SpyC_3_ and then 3B04-SpyTag. Samples were then treated ± light and incubated with HeLa cells to measure apoptosis induction. (e) Beads functionalized with each of the indicated SNAP-PhoCl-SpyC_1–6_ valency engineering covalent capture modules were subsequently functionalized with scFv-SpyTag, washed, and exposed to 405 nm light for 10 min. Samples were centrifuged, and 9 μL of the supernatant was loaded on a denaturing SDS-PAGE gel. (f) Released multivalent assemblies from panel e were incubated with HeLa cells for 2 h at the indicated concentrations. Cells were then assayed for apoptosis induction by incubation with NucView 488 and subsequent flow cytometry. Effective scFv concentration is the concentration of scFv in each well regardless of its multivalent state. Data were obtained in triplicate. The dashed line indicates 50% apoptosis, and the sigmoid curves are fitted Hill equations.

As an extension to the tools already exhibited, we sought to develop a method of releasing captured proteins into solution upon exposure to a specific cue. Ideally, this process would be simple, highly controllable, and stable, without the requirement for addition of further reagents. Recent advances have enabled the use of genetically encoded photocontrollable elements for micropatterning^24^ and control of hydrogel stiffness^60^ utilizing the photocleavable protein PhoCl.^23^ Upon exposure to violet light (405 nm), PhoCl cleaves its own backbone, thus allowing for the controlled release of attached proteins. Previous attempts to use PhoCl for the controlled release of proteins from hydrogels used click chemistry for immobilization, which can negatively affect protein functionality through non-site-specific protein capture as well as limiting the engineerability of the system through a lack of orthogonality and easy modularity.^24^ Therefore, we designed and tested a new modular building block, SNAP-PhoCl-SpyCatcher, that would release the SpyCatcher and any associated cargo from the hydrogel (Figure 6a). Cleavage in solution was first verified, with significant cleavage seen after just 1 min of exposure to light (Figure 6b). Due to the transparent nature of the PHD beads, we expected photocleavage to retain comparable efficiency when the SNAP-PhoCl-SpyCatcher modular building block is captured on-bead. To test this, beads were functionalized with SNAP-PhoCl-SpyCatcher and exposed to 405 nm light. After light exposure (to prevent any effect of photobleaching), beads were incubated with mCherry-SpyTag to assay for PhoCl cleavage and, hence, loss of the SpyCatcher entity from bead. A decrease in mCherry fluorescence thus indicates a release of SpyCatcher from the bead, and after 15 min of exposure to 405 nm light, around 80% of protein is released (36% decrease in fluorescence after 5 min, and 79% after 15 min; Figure 6c). Improved photocleavage proteins, such as the recently developed PhoCl2,^61^ can be easily incorporated based on the modular design.

Many protein–protein interactions rely upon specific valencies of the interacting partners to trigger a specific cellular response.^62,63^ Engineering the valency state of protein-based therapeutics that are designed to drug such biological systems typically relies upon laborious in-frame cloning and expression, limiting the capacity of a researcher to investigate many different drugs at many different valencies. The SpyCatcher technology has already been demonstrated to facilitate valency engineering through the post-translational assembly of monomeric nanobody-SpyTag into multivalent constructs via capture on SpyCatcher-coiled coil domain fusions.^22^ We build upon this work by capturing SpyTag fusion proteins on PHD beads functionalized for valency engineering, thus taking advantage of surface immobilization for washing and handling, and the subsequent release of assay components (e.g., in response to a supplied cue of light) to remove surface effects completely. To this end, we mounted distinct populations of beads with one of six SNAP-PhoCl-SpyCatcher fusion proteins (SNAP-PhoCl-SpyCatcher_1–6_, differing in the number of SpyCatcher repeats). Subsequent incubation with a monomeric SpyTag fusion protein results in assembly into photoreleasable, tunably multivalent constructs, depending only on the SpyCatcher module used (Figure 6a). An anti-TRAIL-R1 scFv^64^ (3B04) was chosen as a candidate for molecular engineering as related scFv TRAIL-R1 agonists^65^ reformatted as IgG had undergone a clinical trial, with no clinical benefit seen in either non-small-cell lung cancer^66^ or colorectal cancer.^67^ TRAIL-R1 is widely considered to signal as a trimer and, *in vivo*, is agonized by the trimeric TRAIL,^68^ and we therefore hypothesized that enhanced potency could be achieved by engineering multivalent versions of the scFv. Similar multivalency engineering approaches have been carried out for nanobodies that target TRAIL-R2, a highly related receptor also found to be overexpressed on cancer cells, with great success,^22,69^ but to our knowledge, no such investigation has been carried out for scFvs targeting TRAIL-R1.

Initially, we investigated the effect of making 3B04 trivalent (Figure 6d) through the incubation of 3B04-SpyTag with beads functionalized with SNAP-PhoCl-SpyCatcher_3_ and the subsequent exposure of half of these beads to 405 nm light. We observed that the trivalent engineered scFv construct is more potent than the monovalent scFv and also that release of the multivalent assembly from the bead surface is necessary to fully induce apoptosis. The lower potency of the on-bead trivalent scFv is likely due to the sequestration of trivalent scFv assemblies within the volume of the bead, inaccessible to the cell surface receptors. This cell exclusion effect is also noted in a study by Abate et al.,^70^ in which yeast cells encapsulated within a microdroplet with a polyacrylamide hydrogel bead only grow in the peripheral aqueous zone between bead and droplet edge. It was straightforward to further engineer the valency state of 3B04-SpyT through incubation with separate bead populations, each functionalized with one of the six valency engineering modules (SNAP-PhoCl-SpyCatcher_1–6_). Subsequent exposure to 405 nm light released each of the fully 3B04-conjugated valency engineering modules into solution with little-to-no underfunctionalized modules being observed in SDS-PAGE gel analysis (Figure 6e). We incubated serial dilutions of each of these constructs with HeLa cells for 2 h and measured apoptosis induction using a fluorogenic caspase-3 substrate (NucView 488; Figure 6f). Importantly, the data are presented normalized to the scFv concentration in the assay (measured by the A^280^ value of the assembled construct and multiplied by the number of scFv molecules captured on an assembly); therefore, the observed shift in potency is due to the effect of different valencies rather than a concentration effect. Decreases in the EC50 values indicate significant increases in potency for all multivalent constructs over monovalent scFv (e.g., >50-fold for the pentavalent versus monovalent format; Table 1). Intriguingly, we observe an approximately 2-fold *reduction* in potency when increasing scFv valency from 4× or 5× to 6×. This notion is consistent with previous observations that TRAIL-R1 signaling is dependent not only on trimerization but also on colocalization of numerous TRAIL-R1 trimers within lipid rafts.^71^ We speculate that the 4× and 5× constructs may promote trimer formation while also forming a lateral “bridge” between consecutive TRAIL-R1 trimers, whereas the 6× construct may only enhance formation of a pair of trimers.

**Table 1:** EC50 Values and Standard Deviations for Multivalent Antibody-Induced Cancer Cell Apoptosis^a^

scFv valency	EC50 (nM)
1	114 ± 18
2	15.8 ± 1.2
3	8.21 ± 0.26
4	2.39 ± 0.17
5	1.99 ± 0.17
6	5.88 ± 0.52

a All EC50 values differ significantly from each other (*p* < 0.005, Welch’s two-tailed t-test). Conditions as per Figure 6f.

## Conclusions and Implications

### Accessible, Personalized Technology Platform for Protein Immobilization

In contrast to commercial microbeads (made of, e.g., polystyrene), PHD beads have user-definable attachment points and therefore bring customizable orthogonality and control over the valency of protein immobilization into the hands of the researcher, who can exert this control in their laboratory simply by modifying the concentration of components in the hydrogel synthesis mixture. This reduces reliance on commercial suppliers; avoids batch-to-batch variation outside the control of the researcher; enables a simple method for delivering user-defined amounts of protein to bioassays; and allows personalized variation of the type of tags used. Furthermore, the simple microfluidic bead synthesis ensures monodispersity at a level of control that is not available for commercial beads, providing flexibility and robustness to bioassays. Attachment points are selective (allowing, e.g., direct purification of the protein from a cell lysate), which is brought about by covalent tagging. In addition to SNAP- and Halo-tag as used in this study, other tags are also available which could further expand the orthogonality and engineerability of this system.^72,73^ The site-specific nature of protein capture minimizes the potential impact of immobilization on the activity of the protein of interest, while the covalent nature ensures that captured proteins remain stably associated with the hydrogel and do not leach into solution. Surface effects that are frequently encountered when proteins are physically immobilized on plastic surfaces are minimized, and hydrogels can be expected to mimic the natural environment for soluble proteins much better than a hydrophobic surface. The 3D distribution of attachment points throughout the hydrogel volume (rather than the surface of commercial microbeads) enables each bead to be decorated with 150 million protein molecules or more (∼1.5 billion for 500 μM BG beads) in contrast with ∼660 thousand protein molecules captured on commercial streptavidin beads (Figure SI3b). Finally, hydrogel beads are optically transparent, so that fluorescent measurements are possible, and a strong signal over background can be detected in all fluorescent channels, while commercial magnetic polystyrene beads exhibit autofluorescence in relevant channels, limiting assay sensitivity.^16^

### Versatile Assay Formatting

Based on the modular design principles of synthetic biology, PHD beads can be decorated by attaching tagged protein constructs in a generic way, in an effectively “plug and play” solution for biological experiments and engineering. This approach mirrors “click chemistry”^18^ by providing universal procedures for attachment that do not have to be adjusted on a case-by-case basis. Direct capture of POIs as SNAP or Halo-tag constructs initially simplifies protein purification directly from cell lysates, and this direct capture can be further augmented by the use of secondary capture modules which enable the expansion of protein capture to endogenous untagged targets (e.g., IgG) through the use of defined recombinant affinity reagents. We have developed a suite of these, focusing on bacterially expressible scaffolds to increase accessibility to the technology, and this suite could be readily expanded through the fusion of other affinity reagents (e.g., DARPins, nanobodies) to SNAP- or Halo-tag via modular cloning strategies. The use of defined, recombinant affinity reagents at the core of the PHD technology satisfies an urgent need to reduce the use of animal-derived, polyclonal reagents (as highlighted, e.g., in recent EU directives^74^). Including secondary covalent capture modules (e.g., SpyCatcher/SpyTag, SnpCatcher/SnpTag) adds an extra layer of stable engineerability to the system and enables a second dimension of orthogonality for the creation of multifunctional hydrogels, while the use of valency-engineering modules allows monomeric proteins to be readily assembled into multivalent constructs. Complex multivalent and/or multiprotein decorations are accessible from (separate or mixed) solutions of monomers—these decorations are assembled on-bead and render cloning of additional multivalent constructs unnecessary. Multivalency^75,76^ and induced proximity^77^ is a natural mechanism of enhancing and manipulating interactions in biological systems by cooperativity,^78^ most prominently in natural antibody biology and the biotherapeutics inspired by it.^82^ There are no general rules for the design of multivalent constructs that take advantage of entropic, avidity, or colocalization effects, so the orientation of monomers has to be empirically explored, and an experimental format to empirically assess the contribution of multivalency is necessary. This fact is highlighted in our work by the most potent inducer of apoptosis being a pentavalent antibody construct, despite knowledge that the target (TRAIL-R1) is agonized by a trimeric ligand *in vivo*. Typically, multivalent constructs are cloned and expressed as in-frame fusion proteins, requiring extensive and often practically difficult cloning (e.g., for sequence-homologous repeats that create PCR problems), alongside often expensive and complex mammalian cell expression (e.g., in the case of IgG), limiting both the accessibility of protein engineering and its throughput. However, with PHD beads, a judicious choice of valency engineering modules can bring about such constructs in multiple permutations simply by incubation instead of cloning, once the monomeric modules are available.

Versatility is further boosted by the possibility of photorelease. Steric hindrance and proximity to an ill-defined or hydrophobic surface can limit the applicability of protein assays on beads (in particular for cell–protein interactions), even though the 3D distribution in PHD beads and the solution-like nature of the hydrogel minimize these effects. However, the feature of controlled release of the bead-displayed proteins by optical control removes this common objection against the use of immobilized proteins in assays (as seen by the release of small molecule compounds in OBOC assays^79^). We show that trivalent scFv has to be released from beads in order to potently induce apoptosis. This observation is consistent with the specific exclusion of cells from the internal volume of the polyacrylamide hydrogel, an effect also observed with yeast cells by Abate et al.,^70^ while still allowing large, biologically relevant macromolecules (e.g., IgG) to permeate fully. This “permeability and exclusion feature” could be taken advantage of, and engineered further, in future applications involving therapeutic protein delivery *in vivo*. For instance, protease sites could be added to modules, enabling the tissue-specific release of sequestered/inactive protein drugs.^80,81^ Other future applications to take advantage of optical release could include, e.g., functional tests with proteins that need to be internalized to target intracellular processes or the control of growth factor presentation for tissue engineering.

**Figure 7:**
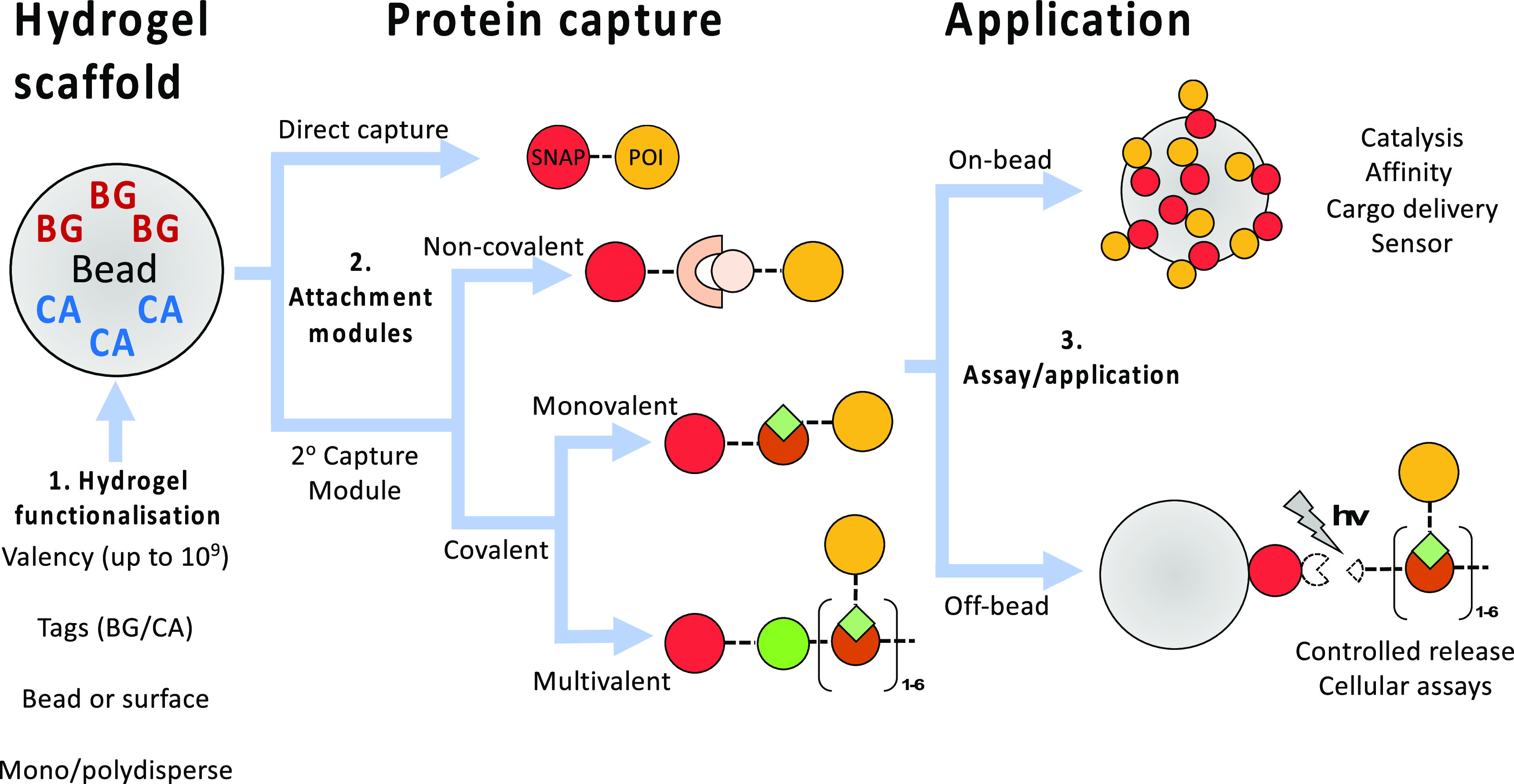
Overview of a modular “build-an-assay” strategy based on PHD beads. Starting from functionalized microbeads (1, see below), choices that define the assay format include the desired valency of each single bead as well as the loading of orthogonal protein capture into the system (controlled by the input concentrations of BG and CA). Next, one can choose how to capture a desired protein (2): either directly as a SNAP- or Halo-tag fusion protein, or via secondary capture modules. Secondary capture modules add the capability to specifically capture native or tagged proteins noncovalently, or to specifically and covalently capture proteins bearing tags, e.g., using the SpyTag-SpyCatcher or SnpTag-SnpCatcher technologies. At this stage, one can also choose to create multivalent constructs from monomeric input proteins of interest through the use of valency engineering modules. Finally (3), the captured proteins can be tested in on-bead assays (e.g., for their affinity) or released from bead in response to irradiation of light, so that the new molecular assemblies can be assayed in solution (e.g., for phenotypic cellular assays). Monodisperse beads can be created in microfluidic devices via water-in-oil emulsions. The design of the microfluidic device and its operation determines the bead size. Alternatively, polydisperse emulsion protocols can be used to make beads at the price of a broader size distribution. As an alternative to the bead format, functionalized hydrogels can also be created on a surface (e.g., for cell culture).

Taken together, the versatility of PHD beads allows an unprecedented degree of freedom in the design of bioassay experiments. Straightforward bead-mediated harvesting of proteins from lysates, valency control (both at the hydrogel decoration stage and for protein constructs), orthogonality of the coupling chemistry (through various tags), and controlled release constitute a technology suite capable of simplifying the planning and execution of discovery campaigns based on modularity (Figure 7). We have demonstrated the simple reformatting of beads and proteins for investigating protein–protein interactions, enzymatic catalysis, bacteriolysis, and phenotypic assays, but an even wider range of assays and applications are conceivable and take advantage of salient features of PHD beads: biocatalysis, *in vivo* drug delivery, controlled release, and sensors.

## Experimental Section

### Protocol for Hydrogel Bead Synthesis and Functionalization

(1) The small molecule anchors (methacrylate-PEG-benzylguanine/methacrylate-PEG-chloroalkane; Table S2.1) for hydrogel functionalization were synthesized by mixing one volume of 40 mM BG-PEG-NH_2_ (NEB S9150S) *or* 40 mM chloroalkane-PEG-NH_2_ (Promega P6741) with one volume of 40 mM methacrylate-NHS (Sigma 730300) overnight at room temperature at 400 rpm in the presence of a 1.5-fold molar excess of triethylamine (Sigma 471283). All solutions were prepared fresh from powder in anhydrous DMSO (Merck 276855) except triethylamine which was added from neat stock. After overnight incubation, the reaction was quenched with 3 volumes of 100 mM Tris-HCl (pH 8.0) and rolled 1 h at room temperature, yielding a final concentration of 5 mM product.
(2) To prepare functionalized beads, unpolymerized hydrogel mix [10 mM Tris-HCl (pH 7.6), 1 mM EDTA, 15 mM NaCl, 6.2% (v/v) acrylamide, 0.18% (v/v) bis(acrylamide), and 0.3% (w/v) ammonium persulfate] containing the small molecule anchors was encapsulated in oil [008- Fluorosurfactant 1.35% w/w, RAN Biotechnologies, and TEMED 0.4% v/v in HFE-7500 (3 M Novec)] in a microfluidic droplet generator (Figure S2.1), as previously described.^27^ After encapsulation, the emulsion was incubated overnight at 65 °C under mineral oil. The next day, polymerized hydrogel beads were recovered by breaking the emulsion with 800 μL of wash buffer (100 mM Tris-HCl, 0.1% Tween-20) and 200 μL of 1*H*,1*H*,2*H*,2*H*-perfluorooctanol (PFO, 97%, Alfa Aesar). The tube was inverted several times and briefly centrifuged for 5 s at 100*g* before recovering the aqueous bead-containing phase into a fresh tube. Large polyacrylamide particles were removed by passing the mixture through a 10 μm filter (CellTrics) for 30 s at 200*g* before using a hemocytometer (KOVA Glasstic) to determine the “concentration” of beads in the suspension. These beads are stable at 4 °C for many months. For all assays, beads are typically incubated and washed in buffer (100 mM Tris-HCl, 0.1% Tween-20). In other buffers and in unbuffered water, the bead pellet after centrifugation can be difficult to identify.
(3) SNAP-tag/Halo-tag fusion proteins were captured by incubating with a defined number of beads for >30 min with rolling in wash buffer. After protein capture, beads were typically washed three times in wash buffer. Subsequent capture of tagged or untagged proteins was performed in the same manner.
(4) On-bead photocleavage was carried out by attaching PCR tubes containing beads to a cooled metal block and exposing to 405 nm light at full power from a LED (M405L2 Thorlabs) driven by LEDD1b (Thorlabs).

## Supporting Information Available

Experimental results (HPLC analysis of BG formation, functionalization of polydisperse PHD beads, valency calculations, cell lysate saturation binding, and modular Gibson assembly design), experimental procedures (benzylguanine analysis via HPLC), microfluidics and bead handling, protein expression and purification, molecular biology of module construction, cell culture, and biological assays (PDF)
